# Effects of Moxa Smoke on Monoamine Neurotransmitters in SAMP8 Mice

**DOI:** 10.1155/2013/178067

**Published:** 2013-05-13

**Authors:** Huanfang Xu, Baixiao Zhao, Yingxue Cui, Min Yee Lim, Ping Liu, Li Han, Hongzhu Guo, Lixing Lao

**Affiliations:** ^1^School of Acupuncture-Moxibustion and Tuina, Beijing University of Chinese Medicine, Beijing 100029, China; ^2^Guang'anmen Hospital, China Academy of Chinese Medical Sciences, Beijing 100053, China; ^3^China & Nanyang Technological University, 50 Nanyang Avenue, Singapore 639798; ^4^Beijing Institute for Drug Control, Beijing 100035, China; ^5^Center For Integrative Medicine, Department of Family and Community Medicine, School of Medicine, University of Maryland, Baltimore, MD 21201, USA

## Abstract

*Objectives*. To investigate the anti-aging effects of moxa smoke on SAMP8 mice. *Methods*. Using 2 × 3 factorial design, exposure length (15 or 30 minutes daily), and concentration (low, 5–15 mg/m^3^; middle, 25–35 mg/m^3^; high, 85–95 mg/m^3^), 70 SAMP8 mice were randomly assigned, *n* = 10/group, to a model group or one of six moxa smoke groups: L_1_, L_2_, M_1_, M_2_, H_1_, or H_2_. Ten SAMR1 mice were used as normal control. Mice in moxa smoke groups were exposed to moxa smoke at respective concentrations and exposure lengths; the model and normal control mice were not exposed. Cerebral 5-HT, DA, and NE levels were determined using ELISA. *Results*. Compared to normal control, the model group showed a significant decrease in 5-HT, DA, and NE. Compared to model group, 5-HT and NE were significantly higher in groups L_2_, M_1_, and M_2_ and DA was significantly so in L_2_ and M_1_. 5-HT, DA, and NE levels were the highest in group M_1_ among moxa smoke groups. A marked exposure length × concentration interaction was observed for 5-HT, DA, and NE. *Conclusion*. Moxa smoke increases monoamine neurotransmitter levels, which varies according to concentration and exposure length. Our finding suggests that the middle concentration of moxa smoke for 15 minutes seems the most beneficial.

## 1. Introduction

Moxibustion, the burning of moxa cones or sticks (made of mugwort, usually *Artemisia vulgaris*) at acupuncture points, is one of the oldest therapies in traditional Chinese medicine (TCM), and it is used for both disease prevention and treatment. During moxibustion, thermal stimulation and moxa smoke are produced simultaneously. The treatment effects of moxa smoke are currently unknown and it is more commonly accepted that thermal stimulation is the key factor for the effects of moxibustion [1]. In fact, it was recorded in the ancient literature that moxa smoke was used to prevent epidemics and it is now used to sterilize the air in hospital wards [2]. Besides, in vitro studies showed that moxa smoke displayed biological activities of tumor-specific cytotoxicity (oral squamous cell carcinoma HSC-2 and HSC-3 and promyelocytic leukemia HL-60) and radical scavenging of O_2_^−^, ^•^OH, ^1^O_2_, and ON [3, 4]. All these strongly indicated that moxa smoke probably had some treatment effects, which contributed to the effect of moxibustion. Moxibustion had been reported to be effective for anti-aging through several ways, including enhancing antioxidant ability by increasing SOD activity and suppressing MDA or NO content and NOS activity [5, 6], enhancing immune function by regulation of serum IL-6 level and IL-2 level [7], and improving neuroendocrine function. As part of the normal aging process, neurotransmitters exhibited a marked alteration in different regions of brain [8, 9], such as reduction of serotonin (5-HT) level in cortex, striatum, and hypothalamus, the dopamine (DA) level in the brain cortex and striatum, and the norepinephrine (NE) content in brain cortex [9]. Since moxibustion showed a good regulation of 5-HT, NE, and DA [10, 11], we hypothesized that moxa smoke may also regulate age-related alteration of monoamine neurotransmitters. In this study, we used an aging animal model senescence-accelerated prone mouse (SAMP8) to study the influence of moxa smoke on the monoamine neurotransmitters 5-HT, DA, and NE in the aging brain. 

## 2. Material and Methods

### 2.1. Animals Preparation

The SAMP8 is the commonly used animal model for aging study and senescence-accelerated mouse/resistance (SAMR1) mice were usually used as a normally aging control for SAMP strains. In SAMP8 mice, senescence naturally occurs four to six months after birth; typical symptoms resemble Alzheimer's disease [12, 13], in which cerebral monoamine neurotransmitters greatly decrease. We obtained 70 SAMP8 and 10 SAMR1 male mice of six months of age and weight of 30 ± 2.7 g from the animal center of the First Hospital Affiliated to Tianjin University of TCM (Tianjin, China). Mice were housed in individual cages with free access to food and water. A controlled environment at a temperature of 20~24°C, humidity of 50%~60%, and 12-hour light-dark cycle was maintained throughout the study. All procedures for animal experiments were conducted in accordance with the World Health Organization's International Guiding Principles for Biomedical Research Involving Animals and were approved by the local ethics committee of Beijing University of Chinese Medicine. 

After acclimation in the animal room for one week, 10 SAMR1 mice were used as normal control, and the 70 SAMP8 mice were randomly assigned (*n* = 10/group) to a model group or to one of the six moxa smoke groups. The six moxa smoke groups were divided into L_1_, L_2_, M_1_, M_2_, H_1,_ or H_2_ using a 2 × 3 factorial design, length of exposure (15 or 30 minutes daily), and moxa smoke concentration (low, 5~15 mg/m^3^; middle, 25~35 mg/m^3^; or high, 85~95 mg/m^3^) (Table 1). The six moxa smoke groups were exposed to moxa smoke six times a week for four weeks; model and normal control mice were not exposed to moxa smoke.

### 2.2. Moxa Smoke Intervention

Custom-designed mouse cages that can accommodate a single mouse and in which the mouse can turn around in the cage and make itself comfortable were used during the moxa smoke interventions. Moxa smoke was generated by burning moxa sticks (three-year-old pure moxa, 0.5 cm × 12 cm, Nanyang Hanyi Moxa Co., Ltd., China). The moxa smoke was contained within a custom-designed glass box (80 cm × 80 cm × 60 cm). Its upper cover, with a circular hole 0.6 cm in diameter, can be shifted. A light-scattering digital dust tester (DT, Beijing BINTA Green Technology Co., Ltd) was used to monitor smoke concentration by detecting levels of PM_10_ (particulate matter < 10 *μ*m in diameter). 

#### 2.2.1. Intervention for the Six Smoke Groups

Groups receiving the same concentration of moxa smoke were exposed together. The main procedure was as follows. The DT was placed in the middle of the glass box to monitor concentration. Mice were individually put into the custom-designed cages, and groups with the same concentration, such as group L_1_ and group L_2_, were put into custom-designed cages, and subsequently, groups with the same concentration were placed on opposite sides of the DT. The burning end of a moxa stick was inserted into the glass box from the upper hole while the other end was held in the investigator's hand. When the box filled with the predetermined amount of smoke, which took about 15, 32, and 82 seconds for low, middle, and high concentration, respectively, the stick was withdrawn and the hole was quickly closed. After 15 minutes, the mice that belonging to the 15-minute exposure group were removed, while the 30-minute exposure group continued for another 15 minutes.

To ensure concentrations of moxa smoke in the specified ranges, moxa smoke concentration was monitored dynamically every three minutes by DT. When the concentration exceeded the upper range, the upper cover of the glass box was moved to release some of the smoke and when it fell below the lower range, the burning moxa stick was reinserted to the box for a few seconds. 

#### 2.2.2. Intervention for the Two Control Groups

The model and normal groups were not exposed to moxa smoke. Mice in those two groups were caged and put on opposite sides of the glass box for 30 minutes with no exposure to moxa smoke.

### 2.3. Cerebral Monoamine Neurotransmitter Assessment

Twenty-four hours after the last intervention, the mice were sacrificed, and cerebrum samples were quickly dissected on an ice board. Using enzyme-linked immunosorbent assay kits of 5-HT, DA, and NE (produced by Nanjing Jiancheng Bioengineering Institute, Nanjing, China), values of absorbance were strictly measured by microplate reader (Multiskan MK3, Finland) at 450 nm wavelength. The concentrations of 5-HT, DA, and NE were determined by comparing the O.D. of the samples to the standard curve.

### 2.4. Statistical Analysis

Data were analyzed by analysis of variance (ANOVA), and post hoc analyses were conducted using the Student-Newman-Keuls test. For that of the six smoke groups, ANOVA for factorial data was used. All values were reported as means ± standard error. Analyses were performed with SPSS software version 13.0; *P* < 0.05 was considered to be statistically significant.

## 3. Results 

### 3.1. Cerebral Monoamine Neurotransmitter in the Normal and Model Groups

**Figure 1:**
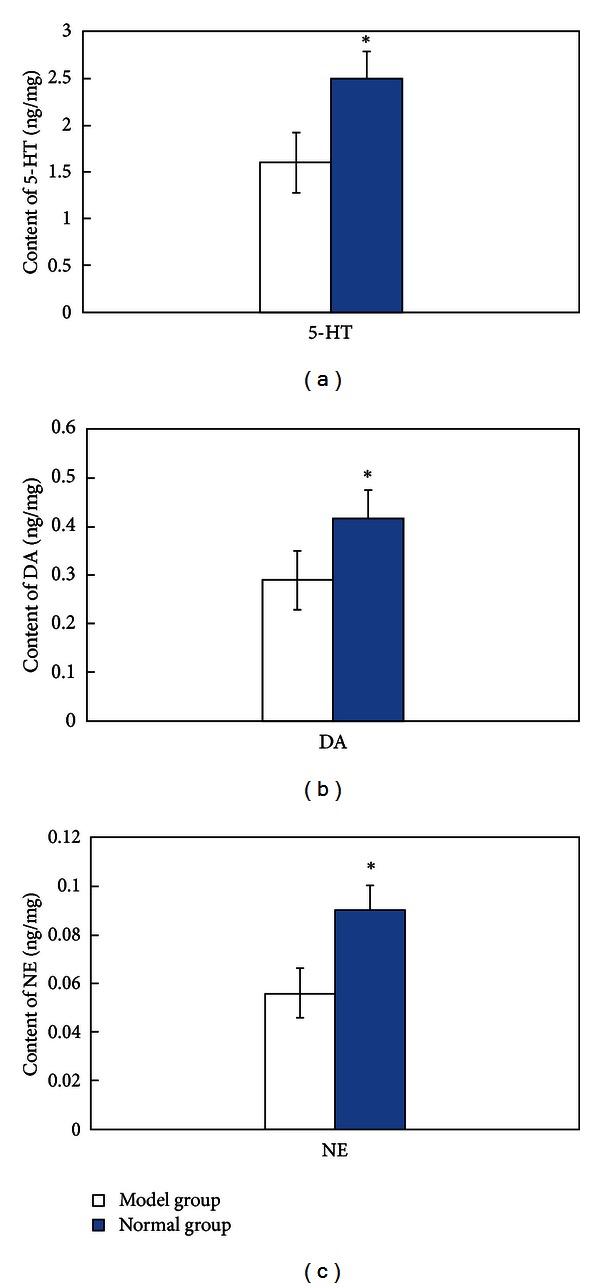
Differences in 5-HT (a), DA (b), and NE (c) levels, normal group (SAMARI mice) versus model group (SAMP8 mice). Note: *P < 0.05, versus model group.

Compared to the normal group, the model group showed a remarkable decrease in cerebral 5-HT, DA, and NE levels (Figure 1).

### 3.2. Effect of Moxa Smoke on Cerebral Monoamine Neurotransmitters in SAMP8 Mice

**Figure 2:**
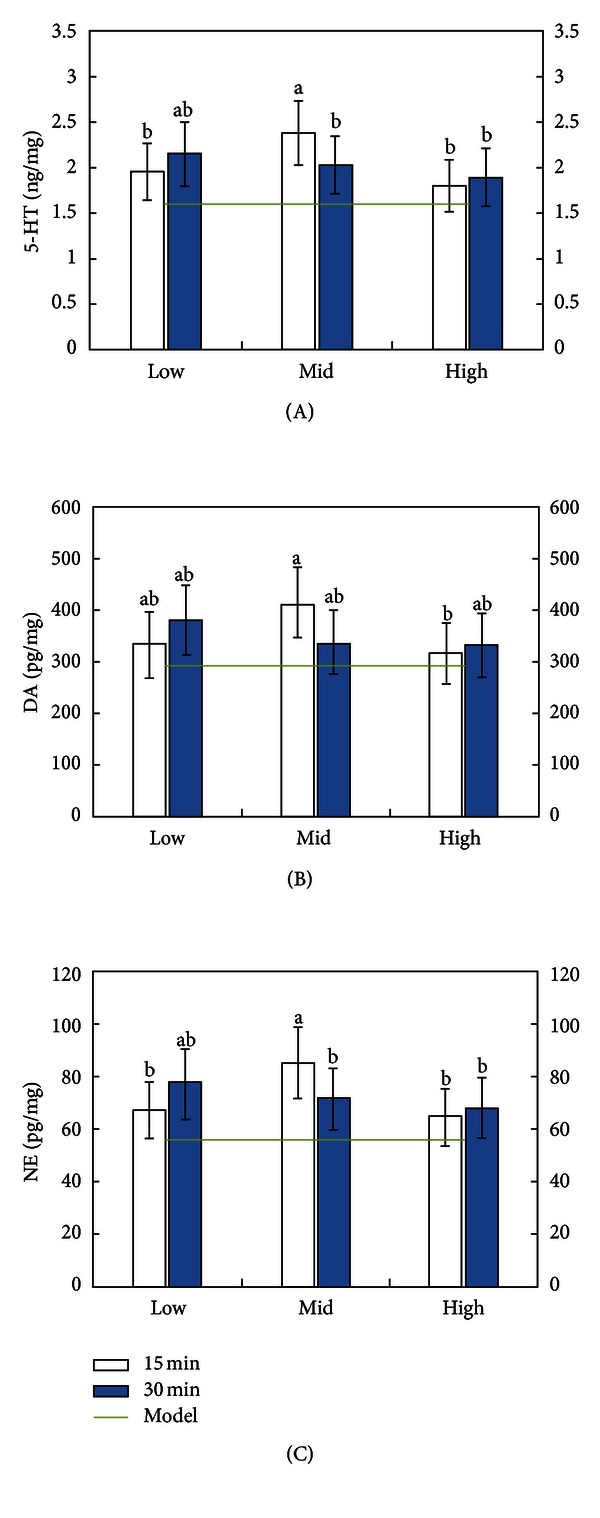
Cerebral levels of 5-HT (A), DA (B), and NE (C) of SAMP8 mice exposed to different concentrations of moxa smoke for various lengths of time. Note: any two groups without a common alphabet (a or b) are significantly different (*P* < 0.05).

Moxa smoke groups showed a higher level of monoamine neurotransmitters than model group. Compared to the model group, 5-HT and NE were significantly increased in L_2_, M_1_, and M_2_, while DA was significantly increased in L_2_ and M_1_ (Figure 2). 

### 3.3. Effects of Concentration and Length of Moxa Smoke Exposure on Cerebral Monoamine Neurotransmitters in SAMP8 Mice

**Figure 3:**
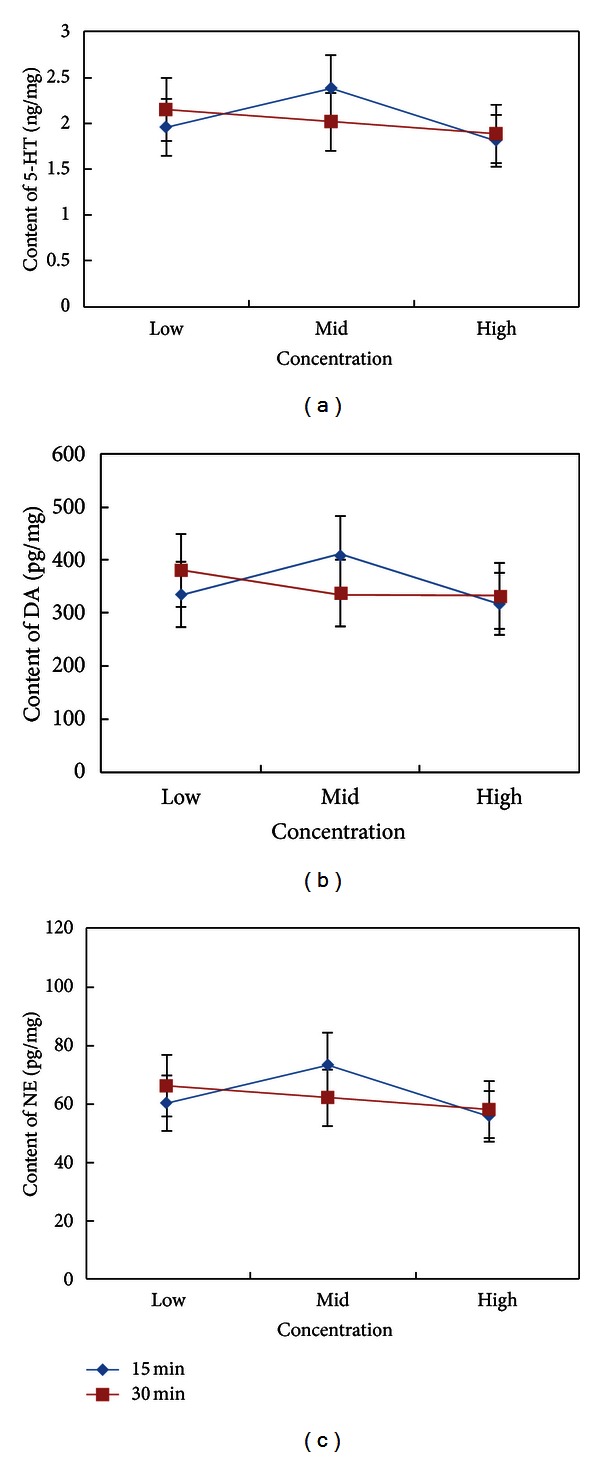
Cerebral levels of 5-HT (a), DA (b), and NE (c) of SAMP8 mice exposed to moxa smoke at different concentrations and for different lengths of time.

Using ANOVA for factorial data, there was a significant interaction between length of exposure and concentration effects on cerebral monoamine neurotransmitter levels (Figure 3). Levels of 5-HT and NE varied significantly as a function of concentration. No main effect of exposure length on monoamine neurotransmitters was found. 

### 3.4. Optimum Conditions for Interventions with Moxa Smoke

Multiple comparisons showed that moxa smoke intervention for the M_1_ group, middle concentration for 15 minutes, manifested the highest effect in increasing cerebral 5-HT, DA, and NE levels among the different combinations between concentration and exposure length (Figure 2).

## 4. Discussion 

In this study, we explored the anti-aging effects of moxa smoke and found that moxa smoke may increase monoamine neurotransmitter levels in SAMP8 mice and the effects were related to exposure length and concentration of moxa smoke. This indicated that moxa smoke may be one of the effective components of moxibustion. 5-HT, DA, and NE were important neurotransmitters in the central nervous system and were closely related to neural functions and aging [14]. More specifically, 5-HT excites the functions of learning and memory [15, 16] and can trigger facilitation, and DA has an excitatory effect on overall behavior and participates in the reappearance of memory trace. NE regulates excitation of the cerebral cortex and influences awakening, sensation, emotions, and advanced cognitive functions; increased excitability of NE improves learning and memory [17]. The SAMP8 mouse is marked by impaired learning and memory. In other studies, six-month-old SAMP8 mice had shown a significant decrease in monoamine and metabolite levels, which was relevant to cognitive impairment, in the cortex and hippocampus [18]. DA levels in the brain had also been shown to decrease with aging, and DA turnover was lower in aged SAMP8 mice than in young ones [19]. Impairment of learning and memory in SAMP8 mice had also been reversed by drugs, and increased cerebral Ach and 5-HT and activation of the PI3 K/AKT pathway were possible mechanisms of this effect [20]. Consistent with those findings, our study showed decreased monoamines in the model SAMP8 mice compared to the SAMR1 mice. Compared to the model mice, SAMP8 mice exposed to moxa smoke showed higher levels of cerebral 5-HT, DA, and NE. This indicated that moxa smoke increased monoamine content, thus postponing senescence. 

Moxa floss is made from mugwort leaf (*Artemisia argyi Folium*), and its smoke contains multiple essential oils, suspended particulate matters, and products of chemical oxidation [21]. The chemical ingredients of moxa floss may lay the foundation for the effects of moxa smoke, such as flavones isolated from *Artemisia argyi* inhibiting proliferation of a couple of tumor cell lines [22] and Arteminolides B-D (2-4) isolated from *Artemisia argyi* inhibiting the farnesyl protein transferase [23]. Clinically, moxa floss with good quality has to be specially processed and preserved for a relatively long time. The active ingredients of mugwort leaves mainly lie in their volatile oil, such as caryophyllene and caryophyllene [24], which can be distilled into moxa smoke. Most of these volatile ingredients are nonaromatic essential oils. Widely used in aromatherapy, they have no fragrance in their natural state but disperse their fragrance when burning. An essential oil can exert an effect via absorption through the skin and through inhalation, and it also can act directly on the central nervous system through the olfactory pathway [25]. Considering the similarity of action between essential oils and moxa smoke, we conjecture that moxa smoke and aromatherapy exert their effects similarly. Further studies concerning the active ingredients of moxa smoke are warranted in order to understand the mechanisms of moxa smoke.

In this study, we also find that the anti-aging effect is linked to smoke concentration, and there is an interaction between concentration and length of exposure. According to our results, monoamine neurotransmitter levels were highest in the M_1_ group (i.e., middling concentration for 15 minutes), which suggested that these may be the optimum specifications for raising monoamine neurotransmitter levels. There is probably a nonlinear relationship between dose (concentration and exposure length) and effect of moxa smoke based on the present results, however; further study is needed to determine the dose-effect curve.

There exist some limitations in this study. Oxygen concentration was not monitored during the intervention procedure. However, the combustion of moxa stick in the glass box was only for a very short duration from 15 to 82 seconds and the glass box was not completely sealed. Methods to monitor the oxygen supply should be applied in subsequent studies. Secondly, behavioral tests of learning and memory should be recommended for future studies. 

In conclusion, our preliminary observation showed that moxa smoke may increase monoamine neurotransmitter in central nerve system and the middle concentration of moxa smoke for 15 minutes seemed most beneficial. However, further investigation to confirm our findings and to explore possible mechanisms of action is warranted. 

## Figures and Tables

**Table 1:** 2 × 3 factorial design.

Factor and level	Concentration		
	Low concentration (5~15 mg/m^3^)	Middle concentration (25~35 mg/m^3^)	High concentration (85~95 mg/m^3^)
Time			
15 min	L_1_	M_1_	H_1_
30 min	L_2_	M_2_	H_2_
